# Ghrelin Protection against Cytotoxic Effect of Ethanol on Rat Salivary Mucin Synthesis involves Cytosolic Phospholipase A_2_ Activation through S-Nitrosylation

**Published:** 2010-03

**Authors:** Bronislaw L. Slomiany, Amalia Slomiany

**Affiliations:** *Research Center, University of Medicine and Dentistry of New Jersey, Newark, NJ, USA*

**Keywords:** ghrelin, ethanol cytotoxicity, salivary mucin, cPLA_2_ activation, S-nitrosylation, cNOS

## Abstract

Recent advances in identifying the salivary constituents of significance to the maintenance of soft oral tissue integrity have brought to focus the importance of a 28-amino acid peptide hormone, ghrelin. Here, we report on the role of ghrelin in countering the disturbances in salivary mucin synthesis caused by ethanol cytotoxicity in rat sublingual gland acinar cells. We show that the countering effect of ghrelin on mucin synthesis was associated with the increase in NO and PGE2 production, and the enhancement in cytosolic phospholipase A_2_ (cPLA_2_) activity. The ghrelin-induced up-regulation in mucin synthesis, like that of cPLA_2_ activity, was subject to suppression by Src inhibitor, PP2, ERK inhibitor, PD98059, as well as Akt inhibitor, SH-5 and ascorbate. Moreover, the loss in countering effect of ghrelin on the ethanol cytotoxicity and mucin synthesis was attained with cNOS inhibitor, L-NAME as well as COX-1 inhibitor, SC-560. Furthermore, while the effect of L-NAME was also reflected in the inhibition of the acinar cell capacity for NO and PGE2 generation, and cPLA_2_ S-nitrosylation, the COX-1 inhibitor caused the inhibition in PGE2 only. Our findings demonstrate that ghrelin protection of the acinar cells against ethanol cytotoxicity and the impairment in salivary mucin synthesis involves Src kinase activation of the Akt/cNOS pathway that leads to up-regulation in cNOS activity. We also show that cNOS-derived NO induction of the cPLA_2_ activation through S-nitrosylation, for the increase in PGE2 generation, is an essential element of the protective mechanism of ghrelin action.

## INTRODUCTION

Among the recognized consequences of alcohol abused on the health of oral cavity are the diminished secretion of saliva, high incidence of microbial and fungal infections, mucosal inflammatory changes, and the increased risk of cancer of the oral cavity (1–3). The salivary gland acinar cell responses to ethanol cytotoxicity are manifested by the elevation in proinflammatory cytokine production, enhancement in apoptosis, impairment in nitric oxide (NO) and prostaglandin signaling pathways, and a marked decrease in salivary protein production (3–6). Moreover, the disturbances in salivary gland mucous acinar cells by ethanol also affect the synthesis of salivary mucins, the glycoproteins implicated in the preservation of the health of oral cavity (7, 8). Indeed, these large highly glycosylated glycoproteins play a major role in viscoelastic properties of saliva, promote bacterial aggregation and clearance from the oral cavity, and participate in the formation of protective coating covering tooth enamel and soft oral mucosal surfaces (8). Hence, the disturbances in the synthesis of salivary mucins caused by ethanol cytotoxicity are directly reflected in the impairment of the protective coating function, thus weakening the inherent resistance of oral mucosa to injury and facilitating the onset of oral disease (7, 8).

Recent advances in identifying the salivary constituents of significance to the maintenance of soft oral tissue integrity have brought to focus the importance of a 28-amino acid peptide hormone, ghrelin (9). This endogenous ligand for the growth hormone secretagogue receptor, produced mainly in the stomach and implicated in the protection of gastric mucosa against injury by ethanol (10–12), has also been identified in oral mucosa as well as saliva and the acinar cells of salivary glands (9). Moreover, ghrelin emerged as an important regulator of the cross-talk between nitric oxide synthase (NOS) and cyclooxygenase (COX) enzyme systems (11, 13), the products of which, NO and PGE2, play direct cytoprotective role in maintaining mucosal integrity along the alimentary tract, including that of oral mucosa (14–16).

Indeed, there are strong indications as to the existence of a functional relationship between the products of NOS and COX systems, as the stimulation of NO production through NOS induction or the exogenous NO donors leads to up-regulation in COX enzymes activation and the increase prostaglandin generation (17–19). The inhibition of NOS, on the other hand, decreases prostaglandin formation (19). Furthermore, studies indicate that the critical event responsible for rapid changes in prostaglandin production is the release of arachidonic acid from membrane phospholipids by the action of cytosolic phospholipase A_2_ (cPLA_2_) enzyme (20, 21). The activity of cPLA_2_ is tightly regulated by post-translational mechanism involving MAPK/ERK-dependent enzyme protein phosphorylation that facilitates the enzyme translocation from cytosol to membrane to gain access to phospholipid substrates (16, 20, 22). Moreover, it has been reported that cPLA_2_ activation and up-regulation in arachidonic acid release for prostaglandin synthesis may also involve the NO-induced enzyme protein S-nitrosylation (23).

In this study, using rat sublingual salivary gland mucous acinar cells, we investigated the influence of ghrelin on the disturbances in salivary mucin synthesis caused by ethanol.

## MATERIAL AND METHODS

### Acinar cell preparation and mucin synthesis

The mucous acinar cells of sublingual salivary gland, collected from freshly dissected rat salivary glands, were suspended in five volumes of ice-cold Dulbecco’s modified (Gibco) Eagle’s minimal essential medium (DMEM), supplemented with fungizone (50 µg/ml), penicillin (50 U/ml), streptomycin (50 µg/ml), and 10% fetal calf serum, and gently dispersed by trituration with a syringe, and settled by centrifugation (16). Following rinsing, the cells were resuspended in the medium to a concentration of 2 × 10^7^ cell/ml, transferred in 1 ml aliquots to DMEM in culture dishes containing [^3^H] glucosamine (100 µ*Ci*), used as a marker of mucin synthesis, and incubated under 95% O_2_ – 5% CO_2_ atmosphere at 37ºC for 16h (24). After washing with DMEM containing 5% albumin to remove free radiolabel, the cells were resuspended in fresh DMEM and incubated for 2h in the presence of 3% ethanol (16). In the experiments evaluating the effect of ghrelin (rat, Sigma), cNOS inhibitor, L-NAME, iNOS inhibitor, 1400W, Src inhibitor, PP2, ERK1/2 inhibitor, PD98059, Akt inhibitor, SH-5 (Calbiochem), COX-1 inhibitor, SC-560, COX-2 inhibitor, NS-398, and ascobate (Sigma), the cells were first preincubated for 30 min with the indicated dose of the agent of the agent or vehicle followed by incubation with ethanol. The viability of cell preparations before and during the experimentation, assessed by Trypan blue dye exclusion assay, was greater than 98%. At the end of the specified incubation period, the cells were centrifuged, washed with phosphate-buffered saline, and the combined supernatants used for mucin assay.

### Mucin analysis and ethanol-induced cytotoxicity assay

The combined cell wash and incubation medium containing ^3^H-labeled mucin were treated at 4ºC with 10 volumes of 2% phosphotungstic acid in 20% trichloroacetic acid for 4h and the formed precipitates were collected by centrifugation. The crude glycoprotein precipitates were dissolved in 6 M urea and chromatographed on a Bio-Gel A-1.5 column, and the mucin fractions eluted in the excluded volume were subjected to analysis for incorporation of radiolabel and protein content (24). For the measurement of ethanol-induced cytotoxicity, the aliquots of cell suspension from the control and various experimental conditions were centrifuged at 300× g for 5 min and the supernatants used for the assay of cytotoxicity using TOX-7 lactate dehydrogenase assay kit (Sigma).

### PGE2 and NO quantification

The aliquots of the acinar cell suspension from the control and various experimental conditions were centrifuged at 1500× g for 5 min and the conditioned medium supernatant collected. PGE2 assays were carried out using a PGE2 EIA kit (Cayman) and 100 µl aliquots of the spent medium supernatant, according to the manufacturer’s instruction. To assess NO production in the acinar cells, we measured the stable NO metabolite, nitrite, accumulation in the culture medium using Griess reaction (25).

### cNOS activity assay

The assay of cNOS activity in the intact salivary gland acinar cells was carried out by measurement of the conversion of L-[^3^H]arginine to L-[^3^H]citrulline. The cells following various experimental conditions were washed with phosphate-buffered saline and incubated at 37°C in HEPES buffer, pH 7.4, containing 124 mM NaCl, 5 mM KCl, 1 mM MgCl_2_, 1.2 mM Cl_2_, 20 mM HEPES, 10 mM glucose, 10mM cold L-arginine, and 3.5 µ*Ci/*ml L-[^3^H]arginine. Each incubation was performed without and in the presence of 400 µM cNOS inhibitor, L-NAME (26). After 15 min, the incubations were terminated with stop solution containing 3mM L-arginine and 4 mM EDTA, the cells were denatured with 0.3 ml of ethanol and extracted with 20 mM HEPES buffer, pH 5.5. The extracts were then subjected to ion exchange chromatography on Dowex 50W-X8 (Na^+^), and the formed l-[^3^H]citrulline contained in the flow through was quantified by liquid scintillation counting. Values obtained in the presence of L-NAME were subtracted from each sample.

### cPLA_2_ activity assay

The measurement of cPLA_2_ activity in the acinar cells following various experimental conditions was carried out using cPLA_2_ assay kit (Cayman). Following experimental treatments, the cells were homogenized in 1 ml of 50 mM HEPES buffer, pH 7.4, containing 1 mM EDTA, centrifuged at 10,000× g for 15 min at 4°C and the supernatants filtered through an Amicon YM30 filter concentrators, followed by 15 min incubation with 5 µM of calcium-independent PLA_2_ inhibitor, bromoenol lactone, and the aliquots (10 µl) of such prepared cell lysates were subjected to cPLA_2_ assay (27).

### cPLA_2_ S-nitrosylation assay

Detection of cPLA_2_ S-nitrosylation was carried out utilizing a biotin switch procedure for protein S-nitrosylation (28, 29). The acinar cells, treated with ghrelin (0.7 µg/ml) or L-NAME (400 µM) + ghrelin and incubated for 2 h in the presence of 3% ethanol, were lysed in HEN lysis buffer and the unnitrosylated thiol groups were blocked with S-methyl methanethiosulfonate reagent (29). The proteins were precipitated with acetone, resuspended in HEN buffer containing 1% SDS, and subjected to targeted nitrothiol group reduction with sodium ascorbate (100 mM). The free thiols were then labeled with biotin and the biotinylated proteins were recovered on streptavidin beads. The formed streptavidin bead-protein complex was washed with neutralization buffer, and the bound proteins were dissociated from streptavidin beads with 50 µl of elution buffer (20 mM HEPES, 100 mM NaCl, 1 mM EDTA, pH 7.7) containing 1% 2-mercaptoethanol (28). The obtained proteins were then analyzed by Western blotting.

### Western blot analysis

The acinar cells, collected by centrifugation, were resuspended for 30 min in ice-cold lysis buffer (16), and following brief sonication the lysates were centrifuged at 12,000 g for 10 min and the supernatants were subjected to protein determination using BCA protein assay kit (Pierce). The samples, including those from biotin switch procedure, were then resuspended in loading buffer, boiled for 5 min, and subjected to SDS-PAGE using 50 µg protein/lane. The separated proteins were transferred onto nitrocellulose membranes, blocked with 5% skim milk, and incubated with the antibody against the phosphorylated protein at 4ºC for 16 h. After 1h incubation with the horseradish peroxidase-conjugated secondary antibody, the phosphorylated proteins were revealed using an enhanced chemiluminescence. Membranes were stripped by incubation in 1 M Tris-HCl (pH 6.8), 10% SDS, and 10 mM dithiotreitol for 30 min at 55ºC, and reprobed with antibody against proteins of interest. Immunoblotting was performed using specific antibodies directed against cPLA_2_, cNOS and phopspho-cNOS (Ser^1177^) (Calbiochem).

### Data analysis

All experiments were carried out using duplicate sampling and the results are expressed as means ± SD. Analysis of variance (ANOVA) was used to determine significance and the significance level was set at P<0.05.

## RESULTS

**Figure 1:**
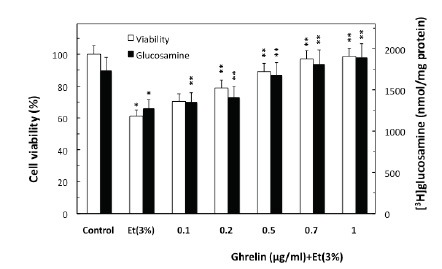
Effect of ghrelin on ethanol-induced cytotoxicity and the synthesis of mucin in rat sublingual salivary gland acinar cells. The cells, labeled with [^3^H]glucosamine as a marker of mucin synthesis, were treated with the indicated concentrations of ghrelin and incubated for 2h in the presence of 3% ethanol (Et). Values represent the means ±SD of five experiments. *P < 0.05 compared with that of control. **P < 0.05 compared with that of Et alone.

**Figure 2:**
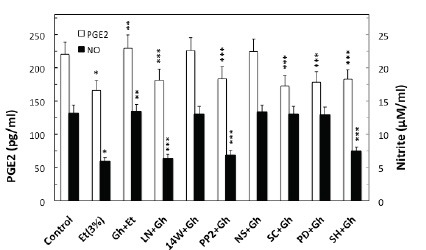
Effect of nitric oxide synthase and cyclooxygenase inhibitors on the ghrelin (Gh)-induced changes in the production of PGE2 and nitrate by sublingual salivary gland acinar cells in the presence of ethanol (Et). The cells, preincubated with 400µM L-NAME (LN), 30µM 1400W (14W), 20µM PP2, 30µM PD98059 (PD), 15µM SC-560 (SC), 20µM NS-398 (NS) or 20µM SH-5 (SH), were treated with 0.7 µg/ml Gh and incubated for 2h in the presence of 3% Et. Values represent the means ± SD of five experiments. *P < 0.05 compared with that of control. **P < 0.05 compared with that of Et alone. ***P < 0.05 compared with that of Gh+Et.

**Figure 3:**
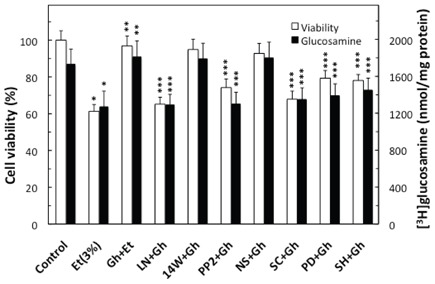
Effect of nitric oxide synthase and cyclooxygenase inhibitors on the ghrelin (Gh)-induced protection against ethanol (Et) cytotoxicity, and the synthesis of mucin in sublingual salivary gland acinar cells. The cells, labeled with [^3^H]glucosamine, and preincubated with 400µM L-NAME (LN), 30µM 1400W (14W), 20µM PP2, 30µM PD98059 (PD), 15µM SC-560 (SC), 20µM NS-398 (NS) or 20µM SH-5, were treated with Gh at 0.7 µg/ml and incubated for 2h in the presence of 3% Et. Values represent the means ± SD of five experiments. *P < 0.05 compared with that of control. **P < 0.05 compared with that of Et alone. ***P < 0.05 compared with that of Gh+Et.

To examine the role of ghrelin in countering the detrimental effect of alcohol abuse on salivary mucin production, we employed rat sublingual salivary gland mucous cells exposed to incubation with ethanol at the dose range (3%) that impairs the acinar cell capacity for mucin synthesis and prostaglandin generation (4–6). We demonstrated that preincubation of the acinar cells with ghrelin led to a concentration-dependent prevention of ethanol cytotoxicity, and resulted nearly complete protection at 0.7 μg/ml of ghrelin (Fig. 1). Moreover, we found that cytotoxicity induced in sublingual salivary gland acinar cells by 3% ethanol was reflected in a 26.2% decrease in mucin synthesis (Fig. 1), as well as a 54.5% drop in NO production and a 24.7% reduction in PGE2 generation (Fig. 2). Ghrelin at its optimal concentration of 0.7 μg/ml for the protection against ethanol cytotoxicity evoked a 42.6% increase in the acinar cell capacity for mucin synthesis (Fig. 3), while PGE2 generation increased by 53.1% and NO production 2.1-fold (Fig. 2).

Further, our results revealed that significant loss in the countering effect of ghrelin on the ethanol-induced toxicity and the acinar cell capacity for mucin synthesis occurred with cNOS inhibitor, L-NAME as well as COX-1 inhibitor, SC-560, while selective iNOS inhibitor, 1400W and a specific COX-2 inhibitor, NS-398 had no effect (Fig. 3). The effect of L-NAME, moreover, was reflected in the inhibition of ghrelin-induced acinar cell capacity for NO production as well as PGE2 generation, whereas the pretreatment COX-1 inhibitor, SC-560, led only to the inhibition in ghrelin-induced PGE2 generation (Fig. 2). The stimulatory effect of ghrelin on the acinar cell capacity for NO and PGE2 production, however, was not affected by the inclusion of iNOS inhibitor 1400W and COX-2 inhibitor, NS-398 (Fig. 2). Moreover, the countering effect of ghrelin on the ethanol-induced changes in the acinar cell capacity for mucin synthesis, and NO and PGE2 production, were subject to suppression by PP2, a selective inhibitor of tyrosine kinase Src, and Akt inhibitor, SH-5. We also revealed that the effect of ghrelin on the acinar cell capacity for mucin synthesis and PGE2 generation was inhibited by MAPK/ERK1/2 inhibitor, PD98059, whereas the production of NO remained unaffected (Figs. 2 and 3).

**Figure 4:**
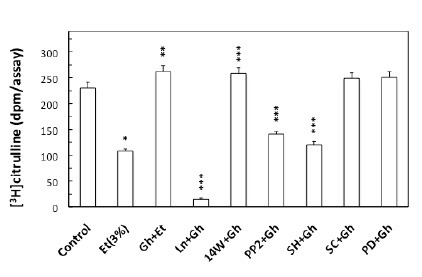
Effect of nitric oxide synthase and cyclooxygenase inhibitors on the ghrelin (Gh)-induced up-regulation in cNOS activity in the acinar cells exposed to ethanol (Et). The cells, preincubated with 400µM L-NAME (LN), 30µM 1400W, 20µM PP2, 20µM SH-5 (SH), 15µM Sc-560 (SC) or 30µM PD98059, were treated with Gh at 0.7 µg/ml and incubated for 2h in the presence of 3%Et. Values represent the means ± SD of five experiments. *P < 0.05 compared with that of control. **P < 0.05 compared with that of Et alone. ***P < 0.05 compared with that of Gh+Et.

**Figure 5:**
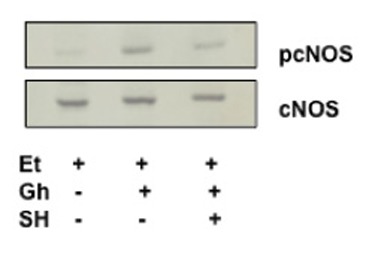
Effect of Akt inhibitor, SH-5 (SH) on ghrelin (Gh)-induced cNOS phosphorylation in sublingual salivary gland cells exposed to ethanol (Et). The cells were treated with Gh (0.7 µg/ml) or SH (20 µM)+Gh and incubated for 2h in the presence of 3% Et. Cell lysates were resolved on SDS-PAGE, transferred to nitrocellulose and probed with phosphorylation specific cNOS (pcNOS) antibody, and after stripping reprobed with anti-cNOS antibody. The immunoblots shown are representative of three experiments.

To gain an additional insight into the involvement of cNOS in ghrelin-induced signaling leading to up-regulation in NO production in response to ethanol, we examined the effect of ghrelin on the acinar cell cNOS activity. We observed that ghrelin countering effect on the ethanol-induced toxicity and the capacity for mucin synthesis was manifested by a 2.4-fold increase in the acinar cell cNOS activity (Fig. 4). Furthermore, the stimulatory effect of ghrelin on the acinar cell cNOS activity was sensitive to L-NAME as well as Src inhibitor, PP2, and Akt inhibitor, SH-5, while iNOS inhibitor, 1400W, ERK1/2 inhibitor, PD98059, and COX-1 inhibitor, SC-560, had no effect (Fig. 4). Further, since rapid cNOS activation is known to occur through enzyme protein phosphorylation by kinase Akt (30), the acinar cells prior to ghrelin incubation, were pretreated with Akt inhibitor, SH-5, and the lysates were examined for cNOS activation using antibody directed against total cNOS and phosphorylated cNOS (pcNOS). As shown in Fig. 5, the countering effect of ghrelin on the ethanol-induced acinar cytotoxicity was reflected in a marked increase in the enzyme protein phosphorylation, while the suppression of ghrelin effect on NO production by Akt inhibition was manifested in a drop in cNOS phosphorylation.

**Figure 6:**
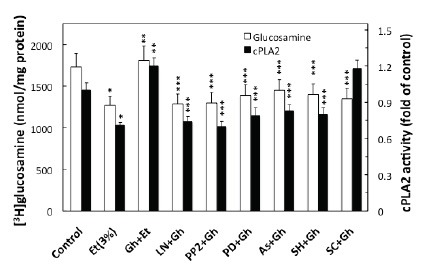
Effect of nitric oxide synthase and cyclooxygenase inhibitors on the ghrelin (Gh)-induced changes in mucin synthesis and cPLA_2_ Activity in the acinar cells exposed to ethanol (Et). The cells, labeled with [^3^H]glucosamine, and preincubated with 400µM L-NAME (LN), 20 µM PP2, 30 µM PD98059 (PD), 300 µM ascorbate As), 20 µM SH-5 (SH) or 15 µM SC-560 (SC), were treated with Gh at 0.7 µg/ml and incubated for 2h in the presence of 3% Et. *P < 0.05 compared with that of control. **P < 0.05 compared with that of Et alone. ***P < 0.05 compared with that of Gh+Et.

We next sought additional leads into the role of ghrelin in regulation of the acinar cell PGE2 generation. As the initial and rate limiting step in prostaglandin production is the liberation of arachidonic acid from membrane phospholipids by highly selective cPLA_2_ (20, 21), we analyzed the effect of ghrelin on the acinar cell cPLA_2_ enzymatic activity. We found that ghrelin countering effect on the ethanol-induced toxicity and the acinar cell capacity for mucin synthesis was reflected in a 68.3% increase in cPLA_2_ activity (Fig. 6). Moreover, the ghrelin-induced up-regulation in cPLA_2_ activity, like that of mucin synthesis, was inhibited by Src kinase inhibitor, PP2 and MAPK/ERK1/2 inhibitor, PD98059, as well as the inhibitor of cNOS, L-NAME, and Akt inhibitor, SH-5. However, COX-1 inhibitor, SC-560, while not causing discernible alteration in the cPLA_2_ activity, exerted the inhibitory effect on mucin synthesis (Fig. 6).

**Figure 7:**
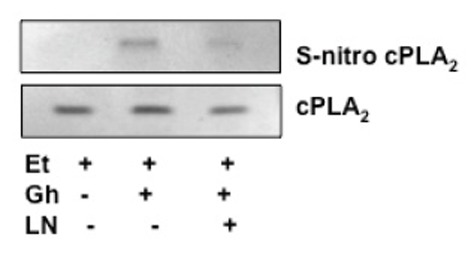
Effect of cNOS inhibitor, L-NAME (LN), on ghrelin (Gh)-induced cPLA_2_ S-nitrosylation in sublingual salivary gland acinar cells exposed to ethanol (Et). The cells were treated with Gh (0.7 µg/ml) or L-NAME (400 µM)+Gh and incubated for 2h in the presence of Et. A portion of the cell lysate was processed by biotin switch procedure for protein S-nitrosylation and, along with the reminder of the lysates, subjected to SDS-PAGE, transferred to nitrocellulose and probed with anti-cPLA_2_ antibody. The immunoblots shown are representative of three experiments.

Finally, we analyzed the influence of S-nitrosylation on the acinar cell cPLA_2_ activity and the capacity for mucin synthesis. The results revealed that ghrelin-induced up-regulation in cPLA_2_ activity, PGE2 generation as well as that of mucin synthesis showed susceptibility to ascorbic acid (Fig.6), which is keeping with known susceptibility of S-nitrosylated proteins to this reducing agent (29, 31). Moreover, Western blot analysis of the acinar cell lysates subjected to biotin switch procedure and probing with antibody against cPLA_2_, revealed that ghrelin prevention of the ethanol-induced cytotoxicity was reflected in the increase in cPLA_2_ protein S-nitrosylation. Preincubation with L-NAME, on the other hand, caused the blockage in the ghrelin-induced cPLA_2_ S-nitrosylation (Fig. 7).

## DISCUSSION

Diminished secretion of saliva, high incidence of microbial and fungal infections, oral mucosal inflammatory changes, and the increased risk of cancer of the oral cavity are well-recognized consequences of alcohol abuse on the health of oral cavity (1–3). The disturbances in salivary gland acinar cell function caused by ethanol cytotoxicity, moreover affect the production of salivary proteins and glycoproteins, including that of mucins, the glycoproteins that play major role in bacterial clearance and the preservation of oral mucosal integrity (3, 5, 7, 8). Hence, in keeping with recent evidence for the involvement of ghrelin in mucosal defense against ethanol toxicity (11–13), in the study presented herein we examined the influence of this 28-amino acid peptide hormone on the impairment in salivary mucin synthesis caused by ethanol.

Employing rat sublingual salivary gland mucous acinar cells exposed to ethanol(4–6), we demonstrated that the protective effect of ghrelin was associated with up-regulation in salivary mucin synthesis, and accompanied by the increase in NO and PGE2 production. Moreover, a significant loss in the countering effect of ghrelin on the ethanol-induced toxicity and the acinar cell capacity for mucin synthesis was attained with cNOS inhibitor, L-NAME as well as COX-1 inhibitor, SC-560, while COX-2 inhibitor, NS-398, and iNOS inhibitor, 1400W had no effect. These results, are thus in keeping with the literature data demonstrating that the detrimental effects of ethanol on salivary mucin synthesis are associated with the impairment in NO and PGE2 generation (3–6, 24), and lend further credence as to the role of ghrelin in regulation of the cross-talk between NOS and COX enzyme systems (11).

Further, we found that the countering effect of ghrelin on the ethanol-induced impairment in mucin synthesis, and NO and PGE2 production, were subject to suppression by Src kinase inhibitor, PP2, whereas the inhibitor of ERK1/2, PD98059, elicited suppression in mucin synthesis and PGE2 generation, but had no effect on NO production. Hence, the activation of tyrosine kinase Src appears to be triggering event whereby ghrelin is capable of affecting the acinar cell capacity for mucin synthesis as well as NO and PGE2 generation. The results also point to the role of MAPK/ERK in the processes of PGE2 generation and mucin synthesis. Moreover, the inhibition of ghrelin-induced acinar cell capacity for mucin synthesis, and NO and PGE2 generation by Akt inhibitor, SH-5, and cNOS inhibitor, L-NAME, and only that of PGE2 and mucin synthesis by COX-1 inhibitor, SC-560, suggest that up-regulation in NO generation through Src kinase activation of the Akt/cNOS pathway plays an essential role in the regulation of PGE2 production and mucin synthesis in response to ghrelin. This interpretation of our results is in concordance with the reports showing that post-translational regulation of cNOS activity occurs through a rapid enzyme protein phosphorylation at the critical Ser^1177^ with the involvement of Src/Akt pathway (16, 26, 32).

Indeed, in our studies, we observed that ghrelin countering effect on the ethanol-induced toxicity and the acinar cell capacity for mucin synthesis was reflected in the increase in cNOS phosphorylation, while the suppression of ghrelin effect of NO production by Akt inhibition was manifested in a drop in cNOS phosphorylation. Moreover, we found that the stimulatory effect of ghrelin on cNOS activity in the intact acinar cells was sensitive to Src kinase inhibitor, PP2 as well as Akt inhibitor, SH-5, but not to ERK1/2 inhibitor, PD98059. Thus, the activation of Src kinase by ghrelin plays an essential role in Akt mediated rapid up-regulation in cNOS activity through phosphorylation.

We next sought additional leads into the role of ghrelin in regulation of the cross-talk between cNOS and COX-1 enzyme systems leading to up-regulation in the acinar cell PGE2 generation. As the initial and rate limiting event in prostaglandin production is the release of arachidonic acid from membrane phospholipids by highly selective cPLA_2_ (16, 20, 21), we analyzed the influence of ghrelin on the processes of cPLA_2_ activation. We found that ghrelin countering effect on the ethanol-induced toxicity was reflected in up-regulation in the acinar cell cPLA_2_ activation, which like that of mucin synthesis and PGE2 generation was subject to suppression by Src kinase inhibitor, PP2, and MAPK/ERK inhibitor, PD98059, as well as the inhibitor of cNOS, L-NAME. However, COX-1 inhibitor, SC-560, while not causing discernible alteration in the cPLA_2_ activity, exerted the inhibitory effect on mucin synthesis. Hence, the activation of the acinar cell cPLA_2_ by ghrelin for the increase in mucin synthesis to counter ethanol cytotoxicity occurs with the involvement of cNOS and requires Src kinase-dependent MAPK/ERK participation. This interpretation of our results is supported by the literature data indicating that post-translational MAPK/ERK-dependent cPLA_2_ phosphorylation on the critical Ser^505^ residue facilitates the enzyme translocation from cytosol to membrane to gain access to phospholipid substrates (16, 20, 22).

While, the post-translational activation through phosphorylation of the key enzymes for prostaglandin and NO generation is well recognized, recent evidence indicates that the increase in prostaglandin formation may also result from NO-induced enzyme protein S-nitrosylation (19, 23, 31). The post-translational activation through S-nitrosylation at the critical cysteine^526^ residue has been linked to the NO-induced enhancement in COX-2 activity (31), while cPLA_2_ activation through S-nitrosylation at cysteine^152^ was reported in human epithelial cells (23). In our study, to assess the role of cNOS-derived NO in the ghrelin-induced cPLA_2_ activation in the acinar cells, we analyzed the influence of S-nitrosylation on the cPLA_2_ activity and the capacity of mucin synthesis. We found that, In keeping with well known susceptibility of S-nitrosylated proteins to reduction by ascorbic acid (28, 29, 31), the ghrelin-induced up-regulation in cPLA_2_ activity and PGE2 generation, as well as the acinar cell capacity for mucin synthesis showed susceptibility to suppression by ascorbate. Furthermore, Western blot analysis of the acinar cell lysates subjected to biotin switch procedure revealed that ghrelin countering effect of the ethanol-induced cytotoxicity was reflected in the increased acinar cell cPLA_2_ protein S-nitrosylation. The suppression of ghrelin effect on NO production with cNOS inhibitor, L-NAME, led to the blockage in the cPLA_2_ protein S-nitrosylation, thus supporting the role of S-nitrosylation in the enzyme activation by ghrelin.

Taken together, our findings demonstrate that the activation of the acinar cell cPLA_2_ through S-nitrosylation plays an essential role in the mechanism of ghrelin protection against the ethanol-induced disturbances in salivary mucin synthesis.
